# TSH and FT4 Reference Interval Recommendations and Prevalence of Gestational Thyroid Dysfunction: Quantification of Current Diagnostic Approaches

**DOI:** 10.1210/clinem/dgad564

**Published:** 2023-09-22

**Authors:** Joris A J Osinga, Arash Derakhshan, Ulla Feldt-Rasmussen, Kun Huang, Tanja G M Vrijkotte, Tuija Männistö, Judit Bassols, Abel López-Bermejo, Ashraf Aminorroaya, Marina Vafeiadi, Maarten A C Broeren, Glenn E Palomaki, Ghalia Ashoor, Liangmiao Chen, Xuemian Lu, Peter N Taylor, Fang-Biao Tao, Suzanne J Brown, Georgiana Sitoris, Lida Chatzi, Bijay Vaidya, Polina V Popova, Elena A Vasukova, Maryam Kianpour, Eila Suvanto, Elena N Grineva, Andrew Hattersley, Victor J M Pop, Scott M Nelson, John P Walsh, Kypros H Nicolaides, Mary E D’Alton, Kris G Poppe, Layal Chaker, Sofie Bliddal, Tim I M Korevaar

**Affiliations:** Department of Internal Medicine, Erasmus University Medical Center, 3000 CA Rotterdam, The Netherlands; Academic Center for Thyroid Diseases, Erasmus University Medical Center, 3000 CA Rotterdam, The Netherlands; Department of Internal Medicine, Erasmus University Medical Center, 3000 CA Rotterdam, The Netherlands; Academic Center for Thyroid Diseases, Erasmus University Medical Center, 3000 CA Rotterdam, The Netherlands; Department of Medical Endocrinology and Metabolism, Copenhagen University Hospital, Rigshospitalet, 2100 Copenhagen, Denmark; Department of Clinical Medicine, Faculty of Health and clinical Sciences, Copenhagen University, 1172 Copenhagen, Denmark; Department of Maternal, Child and Adolescent Health, Scientific Research Center in Preventive Medicine, School of Public Health, Anhui Medical University, 230032 Anhui, China; Department of Public and Occupational Health, Amsterdam UMC, University of Amsterdam, Amsterdam Public Health Research Institute, 1081 HV Amsterdam, The Netherlands; Northern Finland Laboratory Center Nordlab and Medical Research Center Oulu, Oulu University Hospital and University of Oulu, 90570 Oulu, Finland; Maternal-Fetal Metabolic Research Group, Girona Biomedical Research Institute (IDIBGI), Dr. Josep Trueta Hospital, 17007 Girona, Spain; Pediatric Endocrinology Research Group, Girona Biomedical Research Institute (IDIBGI), Dr. Josep Trueta Hospital, 17007 Girona, Spain; Departament de Ciències Mèdiques, Universitat de Girona, 17003 Girona, Spain; Isfahan Endocrine and Metabolism Research Center, Isfahan University of Medical Sciences, 81745-33871 Isfahan, Iran; Department of Social Medicine, School of Medicine, University of Crete, Heraklion, 710 03 Crete, Greece; Laboratory of Clinical Chemistry and Hematology, Máxima Medical Centre, 5504 DB Veldhoven, The Netherlands; Department of Pathology and Laboratory Medicine, Women & Infants Hospital and Alpert Medical School at Brown University, Providence, RI 02903, USA; Harris Birthright Research Center for Fetal Medicine, King’s College Hospital, SE5 9RS London, UK; Department of Endocrinology and Rui’an Center of the Chinese-American Research Institute for Diabetic Complications, Third Affiliated Hospital of Wenzhou Medical University, 325035 Wenzhou, China; Department of Endocrinology and Rui’an Center of the Chinese-American Research Institute for Diabetic Complications, Third Affiliated Hospital of Wenzhou Medical University, 325035 Wenzhou, China; Thyroid Research Group, Systems Immunity Research Institute, Cardiff University School of Medicine, CF10 3EU Cardiff, UK; Department of Maternal, Child and Adolescent Health, School of Public Health, Anhui Medical University, 230032 Anhui, China; Anhui Provincial Key Laboratory of Population Health & Aristogenics, Hefei, 230032 Anhui, China; Department of Endocrinology and Diabetes, Sir Charles Gairdner Hospital, 6009 Nedlands, Perth, Australia; Endocrine Unit, Centre Hospitalier Universitaire Saint-Pierre, Université Libre de Bruxelles (ULB), 1000 Brussels, Belgium; Department of Preventive Medicine, Keck School of Medicine, University of Southern California, Los Angeles, CA 90089, USA; Department of Endocrinology, Royal Devon and Exeter Hospital NHS Foundation Trust, University of Exeter Medical School, EX1 2LU Exeter, UK; Institute of Endocrinology, Almazov National Medical Research Centre, 197341 Saint Petersburg, Russia; World-Class Research Center for Personalized Medicine, Almazov National Medical Research Centre, 197341 Saint Petersburg, Russia; Institute of Endocrinology, Almazov National Medical Research Centre, 197341 Saint Petersburg, Russia; Departament de Ciències Mèdiques, Universitat de Girona, 17003 Girona, Spain; Department of Obstetrics and Gynecology and Medical Research Center Oulu, University of Oulu, 90570 Oulu, Finland; Institute of Endocrinology, Almazov National Medical Research Centre, 197341 Saint Petersburg, Russia; Molecular Medicine, University of Exeter Medical School, Royal Devon & Exeter Hospital, EX3 0AW Exeter, UK; Department of Medical and Clinical Psychology, Tilburg University, 5000 LE Tilburg, The Netherlands; School of Medicine, University of Glasgow, G12 8QQ Glasgow, UK; Department of Endocrinology and Diabetes, Sir Charles Gairdner Hospital, 6009 Nedlands, Perth, Australia; Medical School, University of Western Australia, Crawley, WA 6009, Australia; Department of Women and Children’s Health, Faculty of Life Sciences and Medicine King’s College London, SE5 9RS London, UK; Department of Obstetrics and Gynecology, Columbia University Irving Medical Center, NewYork, NY 10032, USA; Endocrine Unit, Centre Hospitalier Universitaire Saint-Pierre, Université Libre de Bruxelles (ULB), 1000 Brussels, Belgium; Department of Internal Medicine, Erasmus University Medical Center, 3000 CA Rotterdam, The Netherlands; Academic Center for Thyroid Diseases, Erasmus University Medical Center, 3000 CA Rotterdam, The Netherlands; Department of Epidemiology, Erasmus University Medical Center, 3000 CA Rotterdam, The Netherlands; Department of Medical Endocrinology and Metabolism, Copenhagen University Hospital, Rigshospitalet, 2100 Copenhagen, Denmark; Department of Internal Medicine, Erasmus University Medical Center, 3000 CA Rotterdam, The Netherlands; Academic Center for Thyroid Diseases, Erasmus University Medical Center, 3000 CA Rotterdam, The Netherlands

**Keywords:** thyroid gland, thyroid function tests, reference values, pregnancy, thyrotropin, thyroxine

## Abstract

**Context:**

Guidelines recommend use of population- and trimester-specific thyroid-stimulating hormone (TSH) and free thyroxine (FT4) reference intervals (RIs) in pregnancy. Since these are often unavailable, clinicians frequently rely on alternative diagnostic strategies. We sought to quantify the diagnostic consequences of current recommendations.

**Methods:**

We included cohorts participating in the Consortium on Thyroid and Pregnancy. Different approaches were used to define RIs: a TSH fixed upper limit of 4.0 mU/L (fixed limit approach), a fixed subtraction from the upper limit for TSH of 0.5 mU/L (subtraction approach) and using nonpregnancy RIs. Outcome measures were sensitivity and false discovery rate (FDR) of women for whom levothyroxine treatment was indicated and those for whom treatment would be considered according to international guidelines.

**Results:**

The study population comprised 52 496 participants from 18 cohorts. Compared with the use of trimester-specific RIs, alternative approaches had a low sensitivity (0.63-0.82) and high FDR (0.11-0.35) to detect women with a treatment indication or consideration. Sensitivity and FDR to detect a treatment indication in the first trimester were similar between the fixed limit, subtraction, and nonpregnancy approach (0.77-0.11 vs 0.74-0.16 vs 0.60-0.11). The diagnostic performance to detect overt hypothyroidism, isolated hypothyroxinemia, and (sub)clinical hyperthyroidism mainly varied between FT4 RI approaches, while the diagnostic performance to detect subclinical hypothyroidism varied between the applied TSH RI approaches.

**Conclusion:**

Alternative approaches to define RIs for TSH and FT4 in pregnancy result in considerable overdiagnosis and underdiagnosis compared with population- and trimester-specific RIs. Additional strategies need to be explored to optimize identification of thyroid dysfunction during pregnancy.

Optimal maternal thyroid hormone availability is important for facilitating the physiological gestational increase of metabolism as well as the growth and (neuro)development of the fetus. Thyroid function test abnormalities, such as (sub)clinical hypothyroidism, isolated hypothyroxinemia, and (sub)clinical hyperthyroidism have been associated with adverse pregnancy outcomes including gestational diabetes, preterm birth, small for gestational age at birth, and suboptimal neurodevelopment of the offspring (1-6). Thyroid-stimulating hormone (TSH) and free thyroxine (FT4) concentrations considerably change during the course of pregnancy. This is primarily driven by agonistic action of human chorionic gonadotropin on the TSH receptor, changes in thyroid binding proteins, placental type 3 deiodinase expression, and the placental transfer of T4 (7-9). Therefore, reference intervals for nonpregnant individuals are not considered to adequately identify euthyroidism during pregnancy, complicating the diagnosis of thyroid disorders.

Current international guidelines primarily advocate for the establishment of laboratory- and trimester-specific reference intervals for TSH and FT4 (10-12). Despite this primary recommendation being in place for over a decade, there is a lack of systematic data evaluating the diagnostic implications of employing pregnancy-specific reference intervals. Furthermore, practical constraints often preclude the calculation of locally derived reference intervals, necessitating reliance on universal fixed upper limits for TSH and the adoption of nonpregnancy reference intervals for FT4. Several studies have highlighted the pitfalls of employing universal fixed cut-offs, as they tend to culminate in misdiagnoses when applied to diverse local populations (13-15), most likely because TSH and FT4 measurements differ due to various methodologies (assay, preanalytical handling (16)) as well as patient characteristics (body mass index, ethnicity, gestational age (8, 17-19)). However, these investigations were either single-center studies or reliant on aggregated data, limiting their generalizability and applicability for incorporation into guidelines (20). As such, current recommendations of international guidelines on the definition of thyroid dysfunction during pregnancy are largely based on single-center studies and their subsequent extrapolation of physiology (7-13, 21, 22). In order to improve future recommendations and diagnostic policies, robust assessment of the ramifications of current diagnostic approaches is critical, particularly in cases that warrant clinical intervention (eg, clear indication or consideration for medication-based treatment).

In this individual participant data meta-analysis, we aimed to quantify the performance of commonly used alternative diagnostic approaches to laboratory- and trimester-specific reference intervals. These alternatives include (1) use of a fixed upper limit for TSH, (2) employing a modified upper limit of TSH by subtracting from the nonpregnant upper limit of TSH, and (3) utilizing unadjusted nonpregnancy reference intervals for TSH and FT4 as a historical benchmark. We focused on discerning the impact of these alternatives on clinically consequential decisions such as indications or considerations for treatment as per prevailing international guidelines.

## Materials and Methods

### Study Eligibility and Selection

Studies eligible for inclusion were those participating in the Consortium on Thyroid and Pregnancy (https://www.consortiumthyroidpregnancy.org), an international research collaboration dedicated to investigating gestational thyroid (dys)function and its determinants, physiology, and clinical risk profiles. Cohorts included in the consortium are identified through an ongoing systematic review described previously (1). The criteria for inclusion in the current study were prospective population-based cohort studies without selection criteria related to health status with data on TSH, FT4, and thyroid peroxidase antibody (TPOAb) concentrations during the first and second trimesters in pregnancy. We excluded participants with pre-existing prepregnancy thyroid disease, those using thyroid (interfering) medication and those with multiple gestation. Cohorts were excluded if fewer than 120 participants were available after exclusions for reference interval calculations. The study adhered to the Preferred Reporting Items for Systematic Reviews and Meta-Analyses guidelines for Individual Patient Data and we included the preregistered study protocol (CRD42021270078) along with an outline of protocol deviations, which can be found elsewhere (additional material (23)). Study quality and risk of bias were assessed using the Newcastle–Ottawa scale (additional material (23)).

### Defining Reference Intervals, Treatment Indications, and Treatment Considerations

Reference intervals for TSH and FT4 and (the prevalence of) thyroid function test abnormalities (overt and subclinical hypothyroidism, overt and subclinical hypothyroidism with TPOAb positivity, isolated hypothyroxinemia, overt and subclinical hyperthyroidism) were defined uniformly in a cohort-specific manner. Reference intervals were calculated per trimester, defined as <13 weeks, 13 to 27 weeks, and >27 weeks of gestation. For each cohort, trimester-specific TSH and FT4 reference intervals were calculated using the 2.5th to 97.5th percentiles in TPOAb-negative women. TPOAb positivity was defined according to cut-offs provided by the manufacturer. For cohorts with repeated measurements, we used the first available sample for each trimester. Nonpregnancy reference intervals were either published or communicated by the principal investigator of the included cohorts and were assay specific. Information on assays and iodine status per cohort (measured or presumed on the basis of local or international reports) can be found elsewhere (additional materials (23)).

**Figure 1.**
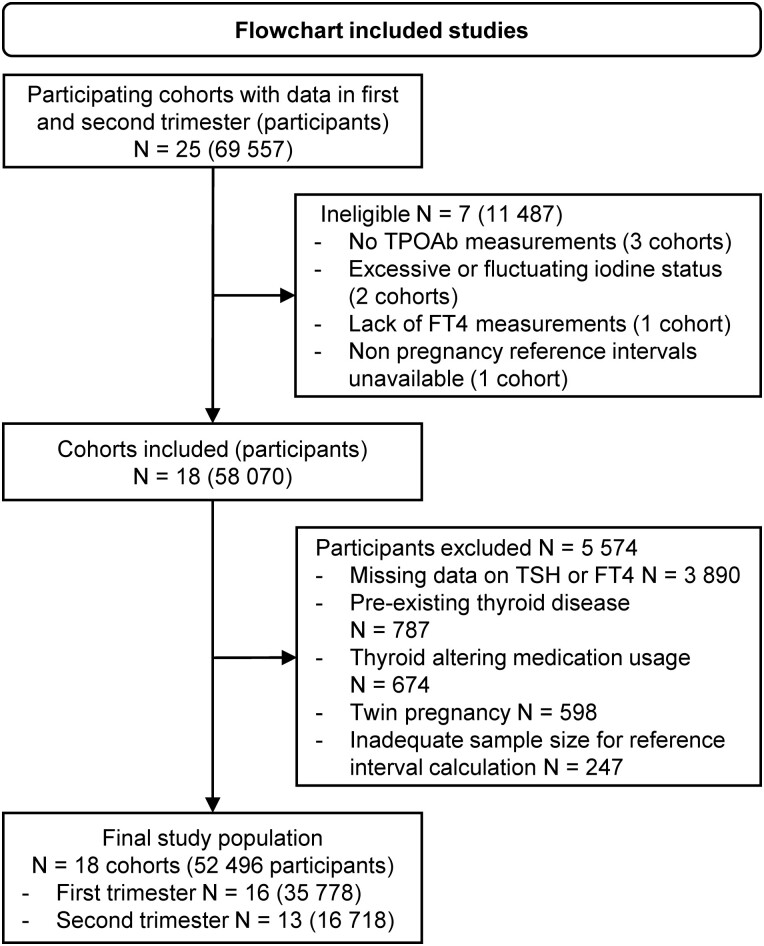
Inclusion flowchart. TPOAb, thyroid peroxidase antibodies.

Thyroid function test abnormalities and prevalences were subsequently defined according to 4 different diagnostic approaches (7) (of which a visual description can be found elsewhere; Figure 1 (23)). Using (1) calculated trimester-specific reference intervals (trimester-specific approach), (2) nonpregnancy reference intervals with a 4.0 mU/L fixed upper limit for TSH (fixed limit approach), (3) nonpregnancy reference intervals with a 0.5 mU/L subtraction from the upper limit of TSH (subtraction approach), (4) unadjusted nonpregnancy reference intervals as a historical benchmark (nonpregnancy approach). Since international guidelines only recommend fixed TSH cut-offs but no fixed FT4 cut-offs, we additionally quantified the role of gestational age–specific FT4 reference intervals by comparing calculated reference intervals as follows: using (5) trimester-specific reference limits for TSH and nonpregnancy reference limits for FT4, and (6) nonpregnancy reference limits for TSH and trimester-specific reference limits for FT4. Treatment indications were defined according to the 2017 American Thyroid Association guidelines; overt hypothyroidism or subclinical hypothyroidism with either a TSH > 10 mU/L or with concomitant TPOAb positivity. A treatment consideration was defined as a TSH between 2.5 mU/L and the upper reference limit with concomitant TPOAb positivity or subclinical hypothyroidism without TPOAb positivity. Treatment of hyperthyroidism was outside the scope of this study, since gestational hyperthyroidism is often considered physiological and we do not have data available to differentiate between gestational transient thyrotoxicosis and Graves hyperthyroidism (10).

The result of each approach was compared to the trimester-specific approach, currently considered the gold standard. Percent stacked bar plots and Sankey diagrams were used to visualize the diagnostic shift, including those between thyroid function test abnormalities, of participants when comparing approaches. A shift in diagnosis was highlighted in the Sankey diagrams (orange flows) when the treatment indication or consideration changed (eg, participants diagnosed with overt hypothyroidism with the reference approach but diagnosed with isolated hypothyroxinemia with the approach investigated).

### Statistical Analyses

Prevalence estimates were aggregated using random intercept logistic regression models, utilizing maximum likelihood to model between-study heterogeneity. This approach was chosen over conventional 2-step inverse-variance approaches due to its preference in sparse event datasets (24, 25). Prediction intervals are presented elsewhere (additional materials to indicate between-study heterogeneity (23, 26)). For each alternative approach, the sensitivity (probability of a positive test result, conditioned on the individual truly being positive) and false discovery rate (proportion of false positives, among positive findings; eg, FDR = FP/(FP + TP)) were calculated compared with the trimester-specific approach. The FDR was chosen over specificity, as it is more sensitive to false positives in instances of sparse outcomes. Outliers were only removed if values were deemed to result from measurement error (outside detectable range; n = 21). All analyses were conducted using R 4.2.2 for Windows (27), employing the meta (28), ggplot2 (29), and ggalluvial (30) packages.

## Results

Out of the 25 cohorts with first and/or second trimester data participating in the Consortium on Thyroid and Pregnancy, 18 fulfilled the eligibility criteria (Fig. 1). After exclusions, the final study population comprised 52 496 participants (Fig. 1) of whom 8.6% were TPOAb positive (range in cohorts 5.7-17.1%). Detailed maternal demographics, cohort-specific prevalences, and an overview of cohort-specific reference limits can be found elsewhere (Tables 1, 2-5, and 6 respectively (23)).

### Prevalences

Pooled prevalences are presented in Table 1 and elsewhere (Table 7 (23)). In the first trimester, the trimester-specific approach was associated with a higher pooled prevalence of total thyroid function test abnormalities than all other approaches (Table 1; Tables 7-8 (23)). The only exception was that a trimester-specific approach was associated with a lower prevalence of subclinical hyperthyroidism (prevalence 1.15%, prediction interval 0.54-2.40) than the alternative methods (prevalence 8.30%, prediction interval 3.60-18.01; Table 8 (23)). In the second trimester, a similar trend could be observed, with higher pooled prevalences for all thyroid function test abnormalities except for subclinical hyperthyroidism (Table 1; Table 8 (23)). In general, heterogeneity was highest for the alternative approaches compared with the trimester-specific approach, reflected by the relatively wide prediction intervals for the alternative approaches (Table 8 (23)).

**Table 1.** Pooled prevalence of gestational thyroid functional test abnormalities according to different reference interval methods

	Treatment indicationPrevalence (CI)	Treatment considerationPrevalence (CI)	Overt hypothyroidismPrevalence (CI)	Overt hypothyroidism and TPOAb+Prevalence (CI)	Subclinical hypothyroidismPrevalence (CI)	Subclinical hypothyroidism and TPOAb+Prevalence (CI)
**First trimester (N = 35 778)**						
Trimester specific approach	1.71% (1.37-2.13)	3.43% (2.91-4.04)	0.51% (0.40-0.64)	0.36% (0.30-0.43)	3.43% (3.14-3.74)	1.24% (0.98-1.57)
4.0 mU/L fixed limit approach	1.19% (0.86-1.65)	3.04% (2.37-3.91)	0.20% (0.09-0.43)	0.13% (0.06-0.28)	2.01% (1.44-2.81)	0.87% (0.66-1.16)
Subtraction approach	1.15% (0.81-1.62)	3.13% (2.39-4.07)	0.22% (0.12-0.39)	0.12% (0.06-0.24)	1.90% (1.28-2.83)	0.82% (0.56-1.19)
Nonpregnancy approach	0.84% (0.57-1.23)	2.87% (2.28-3.62)	0.17% (0.09-0.33)	0.12% (0.06-0.24)	1.19% (0.78-1.82)	0.57% (.37-0.87)
**Second trimester (N = 16 718)**						
Trimester specific approach	1.21% (0.86-1.71)	3.22% (2.94-3.52)	0.31% (0.20-0.47)	0.18% (0.12-0.29)	3.15% (2.84-3.49)	.89% (0.61-1.29)
4.0 mU/L fixed limit approach	1.09% (0.69-1.74)	2.78% (2.04-3.80)	0.33% (0.15-0.69)	0.18% (.09-.37)	1.95% (1.32-2.86)	0.60% (0.37-0.95)
Subtraction approach	1.03% (0.63-1.68)	2.98% (2.17-4.08)	0.36% (0.18-0.74)	0.17% (0.08-0.35)	1.92% (1.11-3.29)	0.56% (0.33-0.93)
Nonpregnancy approach	0.76% (0.44-1.32)	2.63% (1.97-3.50)	0.27% (0.13-0.56)	0.13% (0.06-0.29)	1.16% (0.68-1.97)	0.41% (0.23-0.74)

A treatment indication was defined as either overt hypothyroidism, or subclinical hypothyroidism with TSH > 10 or with concomitant TPOAb positivity, a treatment consideration was defined as a TSH > 2.5 mU/L with concomitant TPOAb positivity or subclinical hypothyroidism without TPOAb positivity.

Abbreviation: TSH, thyroid-stimulating hormone; TPOAb, thyroid peroxidase antibody.

### Diagnostic Performance of Alternative Approaches: Treatment Indication, or Consideration

For identifying women with a treatment indication in the first trimester, a fixed limit approach was associated with a better sensitivity and FDR (0.77 and 0.11) than the subtraction approach (sensitivity 0.74, FDR 0.16) and the nonpregnancy approach (sensitivity 0.60, FDR 0.11; Table 2), but CIs overlapped greatly. Similarly, for identifying women with a treatment consideration in the first trimester, the fixed limit approach (sensitivity 0.70, FDR 0.27) was associated with better pooled estimates than the subtraction approach (sensitivity 0.63, FDR 0.35) and the nonpregnancy approach (sensitivity 0.64, FDR .33; Table 2) while CIs were similar. For the second trimester a similar trend can be observed, with largely overlapping CIs around the diagnostic performance estimates (Table 2).

**Table 2.** Diagnostic performance of different reference interval recommendations as compared to the reference standard

	Treatment indication		Treatment consideration		Overt hypothyroidism		Overt hypothyroidism and TPOAb+		Subclinical hypothyroidism		Subclinical hypothyroidism and TPOAb+	
	Sensitivity	FDR	Sensitivity	FDR	Sensitivity	FDR	Sensitivity	FDR	Sensitivity	FDR	Sensitivity	FDR
**First trimester (N = 35 778)**												
4.0 mU/L fixed limit approach	0.77 (0.58-0.89)	0.11 (0.05-0.22)	0.70 (0.50-0.85)	0.27 (0.14-0.45)	0.55 (0.35-0.74)	0.40 (0.20-0.63)	0.56 (0.38-0.73)	0.41 (0.21-0.64)	0.67 (0.42-0.85)	0.18 (0.11-0.27)	0.69 (0.49-0.84)	0.24 (0.16-0.35)
Subtraction approach	0.74 (0.55-0.87)	0.16 (0.08-0.27)	0.63 (0.46-0.77)	0.35 (0.21-0.52)	0.55 (0.34-0.74)	0.41 (0.23-0.61)	0.56 (0.37-0.74)	0.38 (0.22-0.57)	0.63 (0.37-0.83)	0.23 (0.15-0.35)	0.67 (0.45-0.84)	0.30 (0.20-0.43)
Non pregnancy approach	0.60 (0.42-0.76)	0.11 (0.06-0.19)	0.64 (0.39-0.83)	0.33 (0.18-0.53)	0.49 (0.33-0.65)	0.35 (0.19-0.55)	0.54 (0.37-0.70)	0.38 (0.21-0.58)	0.47 (0.22-0.73)	0.19 (0.12-0.30)	0.55 (0.30-0.78)	0.28 (0.18-0.41)
**Second trimester (N = 16 718)**												
4.0 mU/L fixed limit approach	0.82 (0.65-0.92)	0.24 (0.10-0.47)	0.68 (0.53-0.80)	0.27 (0.17-0.39)	0.84 (0.55-0.96)	0.65 (0.33-0.87)	0.84 (0.55-0.96)	0.65 (0.43-0.82)	0.61 (0.42-0.76)	0.21 (0.11-0.36)	0.65 (0.49-0.78)	0.26 (0.14-0.43)
Subtraction approach	0.82 (0.58-0.94)	0.28 (0.13-0.50)	0.70 (0.52-0.84)	0.34 (0.19-0.53)	0.72 (0.50-0.87)	0.71 (0.37-0.91)	0.82 (0.56-0.94)	0.67 (0.40-0.86)	0.64 (0.39-0.83)	0.26 (0.13-0.46)	0.72 (0.43-0.90)	0.33 (0.19-0.50)
Non pregnancy approach	0.66 (0.45-0.82)	0.29 (0.14-0.49)	0.59 (0.45-0.72)	0.32 (0.20-0.47)	0.69 (0.47-0.84)	0.67 (0.36-0.88)	0.74 (0.53-0.88)	0.64 (0.39-0.83)	0.43 (0.24-0.64)	0.21 (0.10-0.38)	0.52 (0.31-0.72)	0.34 (0.20-0.52)

Data are presented as effect estimate (confidence interval). Reference standard = trimester specific approach, FDR = false discovery rate – proportion of false positive test results among all positive test results. A treatment indication was defined as either overt hypothyroidism, or subclinical hypothyroidism with TSH > 10 or with concomitant TPOAb positivity, a treatment consideration was defined as a TSH > 2.5 mU/L with concomitant TPOAb positivity or subclinical hypothyroidism without TPOAb positivity.

Abbreviations: TSH, thyroid-stimulation hormone; TPOAb, thyroid peroxidase antibody.

### Diagnostic Performances of Alternative Approaches: Thyroid Function Test Abnormalities

In the first trimester, the sensitivity of the alternative approaches to detect either overt or subclinical hypothyroidism or isolated hypothyroxinemia ranged from 0.47 to 0.67 while FDRs ranged from 0.18 to 0.41 (Table 2; Table 9 (23)). In the second trimester, the sensitivity of the alternative approaches was higher for overt hypothyroidism when compared with the first trimester, especially with the fixed limit approach (sensitivity 0.84), although the FDR was also higher (0.65) and CIs overlapped (Table 2). The diagnostic performance of the alternative methods in the second trimester were mostly similar for subclinical dysfunction (Table 2; Table 9 (23)). The diagnostic performance to detect subclinical and overt hyperthyroidism were identical for all alternative approaches, since the lower limit of TSH and the upper limit of FT4 were not varied between alternative approaches. Sensitivity to detect subclinical hyperthyroidism ranged from 0.98 to 1.00 between trimesters while the FDR ranged from 0.76 to 0.90. For overt hyperthyroidism sensitivity ranged from 0.70 to 0.73 and FDRs ranged from 0.46 to 0.55 between trimesters (Table 9 (23)).

### Shift in Biochemical Diagnosis Between Methods

**Figure 2.**
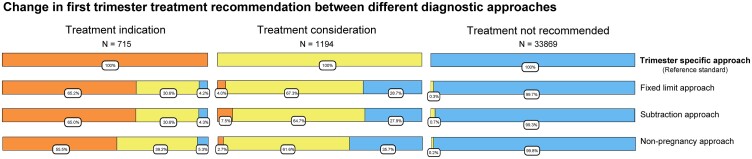
Figure shows participants with a treatment recommendation according to the reference standard (top row, based on trimester-specific reference intervals using 2.5th and 97.5th percentile in TPOAb negative women). Going down the figure shows the proportion of the same group of participants which has a changed treatment recommendation with alternative diagnostic approaches. A treatment indication is defined as overt hypothyroidism, subclinical hypothyroidism with either TSH >10 mU/L or concomitant thyroid peroxidase antibody [TPOAb] positivity). Treatment consideration is defined as TSH between 2.5 mU/L and upper reference limit with positive TPOAb; TSH between RI upper limit and 10 mU/L with negative TPOAb). Fixed limit approach: nonpregnancy reference intervals with a 4.0 mU/L fixed upper limit for TSH. Subtraction approach: nonpregnancy reference intervals with a 0.5 mU/L subtraction from the upper limit of TSH. Nonpregnancy approach: unadjusted nonpregnancy reference intervals as a historical benchmark. All definitions are based on the 2017 American Thyroid Association guidelines.

**Figure 3.**
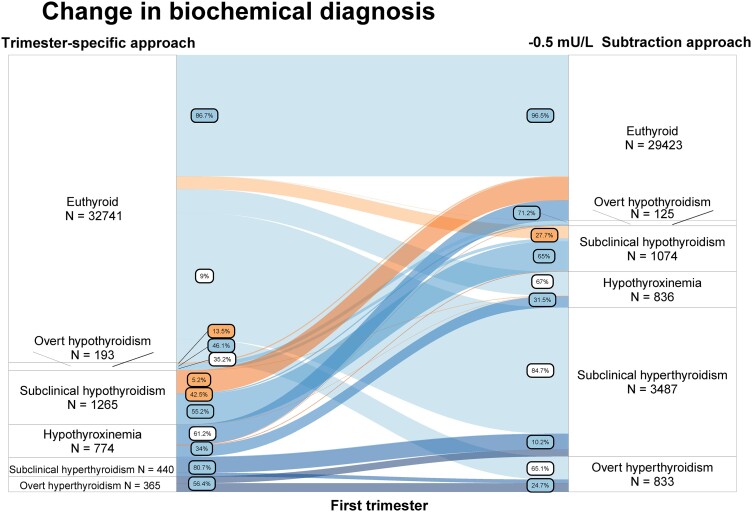
Change in diagnosis comparing the trimester-specific reference intervals (left; using 2.5th and 97.5th percentile in TPOAb-negative women) and the fixed limit approach (right; nonpregnancy reference intervals with a 4.0 mU/L fixed upper limit for TSH). Labels indicate proportion of women for that specific thyroid function test abnormality who change to a certain other label. Orange labels and flow indicate a change in treatment recommendation, white labels indicate a change in biochemical diagnosis but with the same treatment recommendation, blue labels indicate proportion with the same biochemical diagnosis between methods.

**Figure 4.**
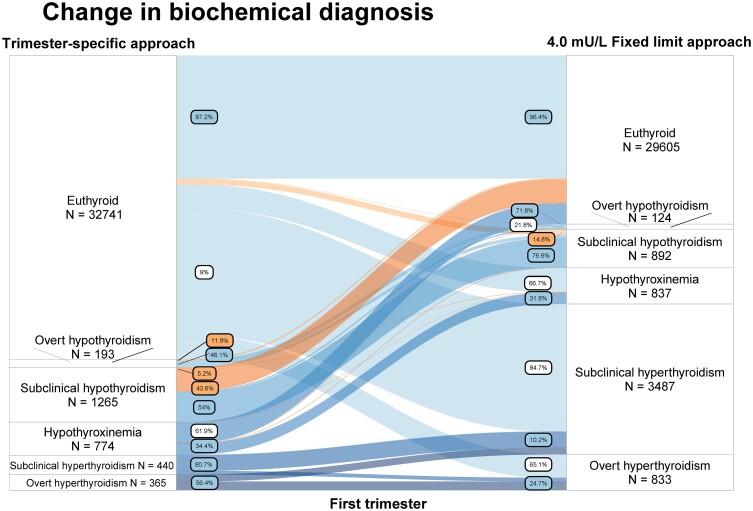
Change in diagnosis comparing the trimester-specific reference intervals (left; using 2.5th and 97.5th percentile in TPOAb-negative women) and the subtraction approach (right; nonpregnancy reference intervals subtracting 0.5 mU/L from the upper limit for TSH). Labels indicate proportion of women for that specific thyroid function test abnormality who change to a certain other label. Orange labels and flow indicate a change in treatment recommendation, white labels indicate a change in biochemical diagnosis but with the same treatment recommendation, blue labels indicate proportion with the same biochemical diagnosis between methods.

The shifts in treatment recommendation and thyroid function test abnormalities when employing different approaches are visualized in Figs. 2-4 and elsewhere (Tables 11-30 (23)) (provided as a benchmark). In the first trimester and compared with the trimester-specific approach, using either the fixed limit approach, the subtraction approach, or the nonpregnancy approach would reclassify 34.9%, 34.8%, and 44.5% of women with a treatment indication to a category without a treatment indication, respectively (30.6%, 30.6%, and 39.2% to a category with a treatment consideration, and 4.2% 4.3%, and 5.3% to a category without a treatment recommendation; Fig. 2; Tables 11, 13, and 15 (23)).

As an example, using the fixed limit approach in the first trimester, out of all women with overt hypothyroidism, 11.9% were reclassified as euthyroid, 36.8% as subclinical hypothyroid, and 5.2% as isolated hypothyroxinemia (Fig. 3; Table 23 (23)). In comparison, with the use of the subtraction approach, out of all women with overt hypothyroidism 13.5% would be reclassified as euthyroid, 35.2% as subclinical hypothyroid, and 5.2% as isolated hypothyroxinemia (Fig. 3; Table 25 (23)). Out of all women with subclinical hypothyroidism in the first trimester, with the use of the fixed limit approach, 43.6% were reclassified as euthyroid; 2.1% as overt hypothyroidism, and 0.2% as isolated hypothyroxinemia (Fig. 3; Table 23 (23)). In comparison, with the use of the subtraction approach, 42.5% were reclassified as euthyroid, 2.1% as overt hypothyroidism, and 0.2% as isolated hypothyroxinemia (Fig. 4; Table 25 (23)). Results for the second trimester for overt hypothyroidism were similar, with the exception that using a fixed limit approach resulted in lower rates of reclassification of overt hypothyroidism to euthyroid compared with the subtraction approach (7.3% vs 9.1% respectively) and isolated hypothyroxinemia (3.6% vs 10.9%, respectively; Tables 24, 26 (23)).

### The Role of Pregnancy and Trimester-Specific FT4 Reference Intervals

Alternative approaches specify an upper limit cut-off for TSH but no limits for FT4, yet diagnoses in clinical practice need to be made using the FT4 concentration as well. Therefore, nonpregnancy FT4 reference intervals are typically used in clinical practice. In the first and second trimester, the combination of nonpregnancy FT4 reference intervals with trimester-specific reference intervals for TSH compared with all trimester-specific reference intervals was associated with sensitivities ranging from 0.97 to 1.00 to detect a treatment indication or consideration, and FDRs ranging from 0.03 to 0.14 (Table 3). In contrast, the use of nonpregnancy reference intervals for TSH resulted in a lower sensitivity (0.65-0.72) to detect both a treatment indication and consideration, and was associated with a higher FDR for a treatment consideration (0.08-0.32; Table 3). For thyroid function test abnormalities in the first trimester, the combination of nonpregnancy FT4 reference intervals with trimester-specific reference intervals for TSH was associated with a sensitivity of 0.62 to detect overt hypothyroidism, 0.59 for isolated hypothyroxinemia, and 0.73 for overt hyperthyroidism, while sensitivity for subclinical hypothyroidism was 0.99 (Table 3; Table 10 (23)). In comparison, when using a trimester-specific FT4 reference interval with a nonpregnancy TSH reference interval, the sensitivity for diagnosing subclinical hypothyroidism was 0.58 and the FDR was 0.07 (Table 3), while the sensitivity was 0.83 for overt hypothyroidism, 0.95 for isolated hypothyroxinemia and 1.00 for both overt and subclinical hyperthyroidism.

**Table 3.** Diagnostic performance of FT4 and TSH nonpregnancy reference intervals

	Treatment indication		Treatment Consideration		Overt hypothyroidism		Overt hypothyroidism and TPOAb+		Subclinical hypothyroidism		Subclinical hypothyroidism and TPOAb+	
	Sensitivity	FDR	Sensitivity	FDR	Sensitivity	FDR	Sensitivity	FDR	Sensitivity	FDR	Sensitivity	FDR
**TSH trimester specific and FT4 nonpregnancy** *^a^*												
First trimester (N = 35 778)	0.97 (0.94-0.98)	0.06 (0.03-0.11)	1.00 (0.98-1.00)	0.03 (0.03-0.05)	0.62 (0.38-0.82)	0.38 (0.17-0.65)	0.63 (0.41-0.81)	0.37 (0.18-0.61)	0.99 (0.95-1.00)	0.09 (0.07-0.11)	0.99 (0.94-1.00)	0.14 (0.10-0.19)
Second trimester (N = 16 718)	0.98 (0.95-0.99)	0.14 (0.07-0.27)	0.99 (0.99-0.99)	0.04 (0.02-0.06)	0.90 (0.57-0.98)	0.61 (0.33-0.83)	0.87 (0.58-0.97)	0.63 (0.41-0.81)	0.98 (0.89-1.00)	0.07 (0.04-0.11)	0.99 (0.80-1.00)	0.14 (0.09-0.22)
**TSH nonpregnancy and FT4 trimester specific** *^b^*												
First trimester (N = 35 778)	0.72 (0.42-0.90)	0.08 (0.04-0.15)	0.65 (0.4-0.84)	0.30 (0.15-0.52)	0.83 (0.66-0.93)	0.14 (0.09-0.23)	0.84 (0.70-0.92)	0.15 (0.09-0.22)	0.58 (0.25-0.86)	0.07 (0.03-0.14)	0.65 (0.34-0.87)	0.12 (0.05-0.24)
Second trimester (N = 16 718)	0.68 (0.45-0.85)	0.17 (0.08-0.33)	0.65 (0.47-0.80)	0.32 (0.20-0.48)	0.81 (0.65-0.91)	0.25 (0.16-0.39)	0.89 (0.75-0.95)	0.33 (0.20-0.49)	0.60 (0.29-0.85)	0.15 (0.06-0.32)	0.64 (0.40-0.83)	0.25 (0.11-0.46)

Data is presented as effect estimate (CI). Reference standard = trimester specific approach, FDR = false discovery rate – proportion of false positive test results among all positive test results.

^*a*^Using trimester specific reference intervals for TSH and nonpregnancy reference intervals for FT4 as a means to quantify sensitivity and FDR due to variation between the trimester specific and nonpregnancy reference intervals of FT4.

^*b*^Similar methodology but vice versa to quantify sensitivity and FDR due to variation between the trimester specific and nonpregnancy reference intervals of TSH. A treatment indication was defined as either overt hypothyroidism, or subclinical hypothyroidism with TSH > 10 or with concomitant TPOAb positivity, a treatment consideration was defined as a TSH > 2.5 mU/L with concomitant TPOAb positivity or subclinical hypothyroidism without TPOAb-positivity.

Abbreviations: FT4, free thyroxine; TPOAb, thyroid peroxidase antibody; TSH, thyroid-stimulating hormone.

## Discussion

Accurately diagnosing thyroid dysfunction in pregnancy remains challenging. While calculation of population- and pregnancy-specific TSH and FT4 reference intervals is considered the optimal approach, this is often not feasible. Our study highlights the suboptimal sensitivity and the FDR that common alternative approaches, such as using a fixed TSH upper limit of 4.0 mU/L or subtracting 0.5 mU/L from the TSH upper limit, have to detect specific thyroid function test abnormalities. Moreover, it is clear from these data that maximizing sensitivity often comes at the cost of a higher FDR, which constitutes a difficult tradeoff. We also identify that the use of nonpregnancy FT4 reference intervals was a primary contributor to diagnostic inaccuracy, especially in the detection of overt hypothyroidism—a condition where prompt management is warranted to mitigate adverse maternal and fetal outcomes (31).

These data provide insights into the extent by which diagnostic accuracy of gestational thyroid function test abnormalities can be influenced by different strategies for defining TSH and/or FT4 cut-offs. This information can be used to weigh the pros and cons of future policy recommendations. An important result from this study is the poor diagnostic accuracy and high FDR with the use of the alternative approaches to identify thyroid function test abnormalities with a treatment indication in the first and second trimester. Two main concepts about the use of alternative approaches arise from these data: (1) The large percentage of overdiagnosis (FDR) in general. While the harms related to unnecessary medicalization and overtreatment are generally difficult to study, they are inevitably present (32). This is particularly relevant for relatively prevalent thyroid function test abnormalities with a high FDR and for whom treatment is either indicated or should be considered, such as subclinical hypothyroid women, making especially this group prone to harm due to suboptimal diagnosis. (2) Clinical studies that assess the risk of adverse outcomes typically use laboratory and trimester-specific TSH and FT4 reference intervals. Therefore, the large diagnostic gap with alternative approaches used in clinical practice makes the generalizability of the results from studies on clinical outcomes likely poor. To verify these 2 concepts, future studies should assess the risk of adverse pregnancy outcomes according to different diagnostic strategies.

Another notable observation was that the diagnostic performance of nonpregnancy TSH and FT4 reference intervals was on average only slightly inferior to recommended alternative strategies with greatly overlapping confidence intervals (eg, TSH upper limit of 4.0 mU/L or 0.5 mU/L subtraction from the nonpregnancy limit). The general trend for the first trimester was that nonpregnancy reference intervals were associated with slightly lower sensitivity and slightly higher FDRs for thyroid function test abnormalities with a treatment indication/consideration compared with alternative approaches. And while the alternative diagnostic recommendations assessed in our study perform suboptimally compared with the reference standard of trimester-specific reference intervals, the concept of implementing modified nonpregnancy reference intervals has some clear advantages. It would be easier to implement worldwide, since nonpregnancy reference intervals are universally available and are laboratory specific, and it could also provide a reference interval for FT4. Furthermore, use of an adaptable rule based on nonpregnancy reference intervals would leave beneficial effects of international laboratory-specific standardization and harmonization efforts intact (33, 34).

Too little attention has been given to the issue that alternative strategies do not include a recommended FT4 reference interval. Interestingly, we identified that the use of a nonpregnancy reference limit for FT4 mainly reduced the accuracy for the diagnosis of overt hypothyroidism, isolated hypothyroxinemia, and (subclinical) hyperthyroidism while the use of a TSH nonpregnancy reference interval reduced accuracy for the diagnosis of subclinical hypothyroidism. While fixed FT4 reference limits cannot be universally recommended due to large interassay differences in absolute FT4 values, our data indicate that a considerable part of the missing diagnostic accuracy could be accounted for by optimizing gestational FT4 reference intervals.

In this study, there were wide prediction intervals for diagnostic accuracy of the alternative approaches. This reflects the large between-study variability of prevalences and diagnostic performance of immunoassays. One reason is the varying sensitivity of various FT4 assays to increased concentrations of thyroxine binding globulin during pregnancy (35, 36). Moreover, another probable reason for interstudy and intrastudy variability is the varying difference between nonpregnancy reference limits, often supplied by the manufacturer and not necessarily reflective of the local population, and the locally derived pregnancy reference limits which are inherently population specific. Thyroid function test–influencing factors such as iodine status or smoking status presumably differ between populations leading to differences in laboratory results. The large between-study variability highlights the challenge for future guidelines to make “a one size fits all” recommendation. Instead, future recommendations could focus on improving local diagnostic assessment rather than defining universally applicable reference limits.

### Strengths and Limitations

To the best of our knowledge, this is the first individual participant data meta-analysis studying the prevalence of thyroid dysfunction in pregnancy according to various commonly used diagnostic approaches. We were able to systematically quantify the consequences of different recommendations related to TSH and FT4 reference intervals as well as diagnosis and prevalence of thyroid dysfunction in pregnancy using a unique individual participant dataset of worldwide prospective cohort studies. Our results are in line with a recent aggregate data meta-analysis which identified the prevalence of thyroid dysfunction in the first trimester (20). We restricted our study to the first and second trimester, since we had only limited data available in the third trimester. Since the majority of clinically meaningful decision making takes place in the first or second trimester, we feel this affected the relevance of the current manuscript only minimally. Furthermore, the results of this study cannot be generalized to populations with iodine deficiency or excess since we only included studies with (presumed) adequate or mild to moderately deficient iodine status. It could be debated that an effect of mild to moderate iodine deficiency on thyroid function test distributions could be present, for instance in the case of local fluctuations in iodine status. However, when meta-analyzing small proportions such as prevalences of thyroid dysfunction, larger numbers of studies per iodine status are required for reasonable power and reliable effect estimates to detect differences between methods. For this reason, stratification by iodine status was not feasible in the current study.

## Conclusion

In conclusion, the current alternative approaches for defining thyroid function reference intervals during pregnancy are markedly inferior than trimester-specific reference intervals. The application of nonpregnancy reference intervals and other alternative approaches yield similar diagnostic inaccuracies. The use of alternative diagnostic recommendations on the methodology to define the upper limit of TSH primarily affected the diagnostic accuracy of thyroid function test abnormalities with a treatment indication/consideration, except for the diagnostic accuracy overt hypothyroidism, which is primarily impacted by recommendations on the methodology to define FT4 reference limits. These results can be used to optimize clinical decision strategies including recommendations made in the setting of clinical guidelines, and for the design of future trials to avoid misinterpretation of relevant thyroid function test abnormalities. The optimal method for simulating trimester-specific reference intervals, however, may very well differ from the current advice. And while individual centers should optimally strive for establishing trimester-specific reference intervals, future efforts should focus on identifying alternative strategies that can identify women with an abnormal thyroid function based on pregnancy-specific reference intervals if these are unavailable.

## Data Availability

The data that support the findings of this study are available from the corresponding author upon reasonable request and with necessary approvals from included cohorts.

## Abbreviations

FDR: false discovery rate
FT4: free thyroxine
RI: reference interval
TPOAb: thyroid peroxidase antibody
TSH: thyroid-stimulating hormone

## References

[1] Korevaar TIM , Derakhshan A, Taylor PN, et al Association of thyroid function test abnormalities and thyroid autoimmunity with preterm birth: a systematic review and meta-analysis. JAMA. 2019;322(7):632‐641. 31429897 10.1001/jama.2019.10931 PMC6704759

[2] Derakhshan A , Peeters RP, Taylor PN, et al Association of maternal thyroid function with birthweight: a systematic review and individual-participant data meta-analysis. Lancet Diabetes Endocrinol. 2020;8(6):501‐510. 32445737 10.1016/S2213-8587(20)30061-9 PMC8168324

[3] Andersen SL , Andersen S, Liew Z, Vestergaard P, Olsen J. Maternal thyroid function in early pregnancy and neuropsychological performance of the child at 5 years of age. Article. J Clin Endocrinol Metab. 2018;103(2):660‐670. 29220528 10.1210/jc.2017-02171 PMC5800834

[4] Jansen TA , Korevaar TIM, Mulder TA, et al Maternal thyroid function during pregnancy and child brain morphology: a time window-specific analysis of a prospective cohort. Lancet Diabetes Endocrinol. 2019;7(8):629‐637. 31262704 10.1016/S2213-8587(19)30153-6

[5] Lee SY , Pearce EN. Testing, monitoring, and treatment of thyroid dysfunction in pregnancy. J Clin Endocrinol Metab. 2021;106(3):883‐892. 33349844 10.1210/clinem/dgaa945 PMC7947825

[6] Toulis KA , Stagnaro-Green A, Negro R. Maternal subclinical hypothyroidsm and gestational diabetes mellitus: a meta-analysis. Endocr Pract. 2014;20(7):703‐714. 24449677 10.4158/EP13440.RA

[7] Dashe JS , Casey BM, Wells CE, et al Thyroid-stimulating hormone in singleton and twin pregnancy: importance of gestational age-specific reference ranges. Obstet Gynecol. 2005;106(4):753‐757. 16199632 10.1097/01.AOG.0000175836.41390.73

[8] Glinoer D , de Nayer P, Bourdoux P, et al Regulation of maternal thyroid during pregnancy. J Clin Endocrinol Metab. 1990;71(2):276‐287. 2116437 10.1210/jcem-71-2-276

[9] Panesar NS , Li CY, Rogers MS. Reference intervals for thyroid hormones in pregnant Chinese women. Ann Clin Biochem. 2001;38(4):329‐332. 11471873 10.1258/0004563011900830

[10] Alexander EK , Pearce EN, Brent GA, et al 2017 Guidelines of the American Thyroid Association for the diagnosis and management of thyroid disease during pregnancy and the postpartum. Thyroid. 2017;27(3):315‐389. 28056690 10.1089/thy.2016.0457

[11] Lazarus J , Brown RS, Daumerie C, Hubalewska-Dydejczyk A, Negro R, Vaidya B. 2014 European thyroid association guidelines for the management of subclinical hypothyroidism in pregnancy and in children. Eur Thyroid J. 2014;3(2):76‐94. 25114871 10.1159/000362597 PMC4109520

[12] De Groot L , Abalovich M, Alexander EK, et al Management of thyroid dysfunction during pregnancy and postpartum: an endocrine society clinical practice guideline. J Clin Endocrinol Metab. 2012;97(8):2543‐2565. 22869843 10.1210/jc.2011-2803

[13] Li CY , Shan ZY, Mao JY, et al Assessment of thyroid function during first-trimester pregnancy: what is the rational upper limit of serum tsh during the first trimester in Chinese pregnant women? J Clin Endocr Metab. 2014;99(1):73‐79. 24276458 10.1210/jc.2013-1674

[14] Osinga JAJ , Derakhshan A, Palomaki GE, et al TSH And FT4 reference intervals in pregnancy: a systematic review and individual participant data meta-analysis. J Clin Endocrinol Metab. 2022;107(10):2925‐2933. 35861700 10.1210/clinem/dgac425 PMC9516198

[15] Bliddal S , Boas M, Hilsted L, Friis-Hansen L, Tabor A, Feldt-Rasmussen U. Thyroid function and autoimmunity in Danish pregnant women after an iodine fortification program and associations with obstetric outcomes. Eur J Endocrinol. 2015;173(6):709‐718. 26315374 10.1530/EJE-15-0358

[16] Springer D , Bartos V, Zima T. Reference intervals for thyroid markers in early pregnancy determined by 7 different analytical systems. Scand J Clin Lab Invest. 2014;74(2):95‐101. 24625026 10.3109/00365513.2013.860617

[17] Korevaar TIM , Medici M, De Rijke YB, et al Ethnic differences in maternal thyroid parameters during pregnancy: the generation r study. Article. J Clin Endocrinol Metab. 2013;98(9):3678‐3686. 23836936 10.1210/jc.2013-2005

[18] Pop VJ , Biondi B, Wijnen HA, Kuppens SM, Lvader H. Maternal thyroid parameters, body mass index and subsequent weight gain during pregnancy in healthy euthyroid women. Clin Endocrinol (Oxf). 2013;79(4):577‐583. 23445086 10.1111/cen.12177

[19] Andersen SL , Andersen S, Carle A, et al Pregnancy week-specific reference ranges for thyrotropin and free thyroxine in the north Denmark region pregnancy cohort. Thyroid. 2019;29(3):430‐438. 30734656 10.1089/thy.2018.0628

[20] Dong AC , Stagnaro-Green A. Differences in diagnostic criteria mask the true prevalence of thyroid disease in pregnancy: a systematic review and meta-analysis. Thyroid. 2019;29(2):278‐289. 30444186 10.1089/thy.2018.0475

[21] Marwaha RK , Chopra S, Gopalakrishnan S, et al Establishment of reference range for thyroid hormones in normal pregnant Indian women. BJOG. 2008;115(5):602‐606. 18333941 10.1111/j.1471-0528.2008.01673.x

[22] Yan YQ , Dong ZL, Dong L, et al Trimester- and method-specific reference intervals for thyroid tests in pregnant Chinese women: methodology, euthyroid definition and iodine status can influence the setting of reference intervals. Clin Endocrinol (Oxf). 2011;74(2):262‐269. 21044115 10.1111/j.1365-2265.2010.03910.x

[23] Osinga JAJ , Derakhshan A, Korevaar TIM. Data from: reference intervals. Consortium on thyroid and pregnancy. Updated 18-07-2023. https://www.consortiumthyroidpregnancy.org/referenceintervals

[24] Lin L , Chu H. Meta-analysis of proportions using generalized linear mixed models. Epidemiology. 2020;31(5):713‐717. 32657954 10.1097/EDE.0000000000001232 PMC7398826

[25] Stijnen T , Hamza TH, Ozdemir P. Random effects meta-analysis of event outcome in the framework of the generalized linear mixed model with applications in sparse data. Stat Med. 2010;29(29):3046‐3067. 20827667 10.1002/sim.4040

[26] Riley RD , Higgins JP, Deeks JJ. Interpretation of random effects meta-analyses. BMJ. 2011;342(feb10 2):d549. 21310794 10.1136/bmj.d549

[27] R Core Team R . A Language and Environment for Statistical Computing. R Foundation for Statistical Computing; 2022.

[28] Balduzzi S , Rucker G, Schwarzer G. How to perform a meta-analysis with R: a practical tutorial. Evid Based Ment Health. 2019;22(4):153‐160. 31563865 10.1136/ebmental-2019-300117 PMC10231495

[29] Wickham H . ggplot2: Elegant Graphics for Data Analysis. Springer-Verlag; 2009.

[30] Brunson JC , Read QD. ggalluvial: Alluvial Plots in ‘ggplot2'. R package version 0.12.3. http://corybrunson.github.io/ggalluvial/

[31] Krassas GE , Poppe K, Glinoer D. Thyroid function and human reproductive health. Endocr Rev. 2010;31(5):702‐755. 20573783 10.1210/er.2009-0041

[32] Korevaar TIM , Medici M, Visser TJ, Peeters RP. Thyroid disease in pregnancy: new insights in diagnosis and clinical management. Nat Rev Endocrinol. 2017;13(10):610‐622. 28776582 10.1038/nrendo.2017.93

[33] Thienpont LM , Van Uytfanghe K, De Grande LAC, et al Harmonization of serum thyroid-stimulating hormone measurements paves the way for the adoption of a more uniform reference interval. Clin Chem. 2017;63(7):1248‐1260. 28522444 10.1373/clinchem.2016.269456

[34] Thienpont LM , Van Uytfanghe K, Van Houcke S, et al A progress report of the IFCC committee for standardization of thyroid function tests. Eur Thyroid J. 2014;3(2):109‐116. 25114874 10.1159/000358270 PMC4109515

[35] Grebe SKG . Laboratory testing in thyroid disorders. In: Luster M Duntas LH and Wartofsky L, eds. The Thyroid and its Diseases: a Comprehensive Guide for the Clinician Springer International Publishing; 2019:129‐159.

[36] Feldt-Rasmussen U . Laboratory measurement of thyroid related hormones, proteins and autoantibodies in serum. In: Kopp DCP, ed. Werner and Ingbar's The Thyroid: A Fundamental and Clinical Text Lippincott Williams & Wilkins; 2021:267‐300.

